# Three datasets reporting unexpected events for everyday scenarios: Over 9000 events human-labelled for overall valence/sentiment, topic category, and relationship to the initial goal of the scenario

**DOI:** 10.1016/j.dib.2021.106935

**Published:** 2021-03-03

**Authors:** Molly S. Quinn, Mark T. Keane

**Affiliations:** a School of Computer Science, University College Dublin, Ireland; b Insight Centre for Data Analytics, University College Dublin, Ireland

**Keywords:** Valence, Sentiment, Unexpected events, Goals

## Abstract

The three datasets described in this paper were collected from online experiments distributed via Prolific.co participant system. Together, the three datasets comprise 9720 text responses of unexpected events participants predicted for everyday scenarios such as going shopping or preparing breakfast. Each event was labelled by at least two independent, human raters on their topic or category (relative to their initial scenario), the valence or sentiment of the event, and whether or not the event mentions words related to the goal stated in the initial scenario. We also include summary data from a pre- and post-test conducted in the course of these experiments, as well as the analysis code in the form of Jupyter Notebooks. We provide this data and relevant code for transparency and reproducibility alongside our *Cognition* paper. The dataset could be useful in training machine learning models on valence/sentiment of everyday unexpected events.

## Specifications Table

**Table:** 

Subject	Psychology
Specific subject area	This data comprises everyday scenarios and the unexpected events that participants generated for those scenarios.
Type of data	Tables Labelled Datasets Jupyter Notebooks
How data were acquired	Data were collected from a crowdsourced experiment on Prolific.co participant recruitment system.
Data format	Processed Data Filtered and labelled data: .csv files Python Code
Parameters for data collection	Data were collected online through the Prolific.co. Participation was limited to United States, United Kingdom, and Irish nationals, who were above the age of 18 at the time of the studies, and whose first language was English. Materials used in the collection of responses are included in the database.
Description of data collection	Participants on Prolific.co participant recruitment system responded to everyday scenarios such as going shopping with an unexpected event they thought might occur next. Responses were recorded using free-response long-essay text boxes in SurveyGizmo. Responses were then labelled by at least two independent raters and any disagreements were resolved through discussion (agreement was very high). Importantly, no participant who participated in any study was allowed to participate in subsequent studies in this series.
Data source location	Countries: United States, United Kingdom, and Ireland
Data accessibility	Repository name: Mendeley Data Data identification number: 10.17632/kkt999sn7b.1 Direct URL to data: http://dx.doi.org/10.17632/kkt999sn7b.1
Related research article	Quinn, M. S., Campbell, K. & Keane, M. T. (2021). Do we “fear for the worst” or “hope for the best” in thinking about the unexpected?: Factors affecting the valence of unexpected outcomes reported for everyday scenarios. *Cognition, 208.*https://doi.org/10.1016/j.cognition.2020.104520

## Value of the Data

• To our knowledge, this dataset comprises the only systematic collection of unexpected events reported by people for everyday scenarios.
• These data and related code are made available for the transparency and reproducibility of the analyses found in the associated paper [1].
• We also hope that these labelled text responses will be useful for training machine learning models on sentiment, text generation in line with prior goals, and unexpectedness of everyday events.

## 1 Data Description

The cognitive sciences have often concerned themselves with how people think about the unexpected. However, most of this research relies on theory-driven definitions of unexpectedness (e.g., low probability events), rather than simply asking people what they think when they “think of the unexpected”. However, the literature of the negativity bias leads us to some initial assumptions about general cognition of unexpected events: unexpected events are more often reported as being negative [2] and, reciprocally, negative events can seem more unexpected, diverse, and complex than positive events [3,4]. To assess people's beliefs about everyday unexpected events, we asked participants of three studies to read short scenarios describing everyday events such as going shopping or preparing a meal and to respond with the unexpected event that they thought happened next.

As an instructional manipulation, we asked some participants to respond with unexpected and good events and others to respond with unexpected and negative events. In a third experiment, we manipulated the valence of the scenarios themselves so that each scenario had a positive and negative version. The effect of valence on generated unexpected events is detailed in our associated Cognition paper [1] but we believe this data has further utility in exploring human cognition of events, as well as psychological and computational theories of how sentiment is used in event generation.

In this data paper, we describe the experimental design of the three main experiments, one pre-test, and one post-test. We attach both raw and processed data, as well as the analysis code used for the associated paper. A list of files and their contents are listed below, and a list of variable names with detailed descriptions is included as a separate document.

I. Folder: 0_data/

**Table:** 

Folder	File Name	Description
0_Experiment1/	expt1_master_post_resolve.csv	Processed data and labels for Experiment 1, post consensus, as a single file
1_Experiment2/	alan_plane.csv anna_interview.csv belinda_meeting.csv bill_holiday.csv bob_job.csv edith_exam.csv john_party.csv karen_bus.csv katie_kitten.csv louise_shopping.csv lucy_loan.csv mary_food.csv michael_tea.csv peter_college.csv rebecca_swimming.csv robert_essay.csv sally_wine.csv sam_driving.csv sean_call.csv steve_gardening.csv	Processed data and labels for Experiment 2, post consensus, as individual files by material
2_PostTest/	expt1_posttest.csv	Processed data for the PostTest, as frequencies of each valence category per material
	post_test_responses.csv	contains the individual responses made to each material in the post-test
3_Experiment3/	expt3_master_post_resolve.csv	Processed data and labels for Experiment 3, post consensus, as a single file
	expt3_pretest.csv	Processed data from the pre-test vetting of materials used in Experiment 3 as frequencies of each valence category per material per condition

II. Folder: 1_code/

**Table:** 

File Name	Description
0_Experiment1.ipynb	Jupyter Notebook (Python code) comprising the descriptive and statistical analysis related to Experiment 1
1_Experiment2.ipynb	Jupyter Notebook (Python code) comprising the descriptive and statistical analysis related to Experiment 2
2_PostTest.ipynb	Jupyter Notebook (Python code) comprising the descriptive and statistical analysis related to the post-test
3_Experiment3.ipynb	Jupyter Notebook (Python code) comprising the descriptive and statistical analysis related to Experiment 3

III. Folder: 2_pipeline/

**Table:** 

File Name	Description
expt1_master_data.csv	the cleaned master data file used for analyses in Experiment 1, created in the course of running code in 0_Experiment1.ipynb
expt2_master_data.csv	the cleaned master data file used for analyses in Experiment 2, created in the course of running code in 1_Experiment2.ipynb

IV. Folder: 3_materials/

**Table:** 

File Name	Description
Expt1_Materials.docx Expt1_Labels.xlsx	Materials and labels used in Experiments 1, 2, and the Post-Test
Expt3_Materials.xlsx Expt3_Labels.xlsx	Materials and labels used in Experiment 3

## 2 Experimental Design, Materials and Methods

### 2.1 Experiment 1

In Experiment 1, participants were recruited from the Prolific.co system and completed the study online. Participants were asked to read 20 everyday scenarios and were asked for an unexpected event that they thought might have occurred next. Each scenario was described in three sentences: (i) a goal-setting sentence, (ii) a sentence with some additional information about the scenario that was *not* an action on the main path to the goal and (iii) a final sentence describing some action that was a plan-step in achieving the goal.

Participants saw the sentences in the above order or with the last two sentences switched, so that they saw the goal-setting sentence, the plan-step, and then the additional information. In our initial analyses, we did not find this manipulation to have any effect on responses. Before responding to the main question, participants were asked a simple question about the scenario they had just read, to ensure that they had read and understood it.

Participants were grouped into four conditions, the key manipulation varied the instructions used in each of the four conditions: the Unexpected (“Then, something unexpected occurred. What do you think happened?”), Unexpected-Good (“Then, something unexpected and good occurred. What do you think happened?”), Unexpected-Bad (“Then, something unexpected and bad occurred. What do you think happened?”) and Goal-Fail conditions (in which the goal stated in the first sentence was negated, and participants were asked “What do you think happened?”).

After data collection, the participant's responses were labelled, by material, according to answer category, valence, and goal-objects used in the response text. Three independent raters did the labelling with high inter-rater reliability, and any disagreements were resolved by taking the majority label assigned. There were no cases of three-way splits.

Labelled data for each of the materials was then combined again and analysed as in the associated paper [1]. The Jupyter Notebook files in this database contain the analysis code used for the results in the paper.

### 2.2 Experiment 2

Participants in Experiment 2 were also recruited through the Prolific.co system and completed the study online. Participants read the same scenarios as those used in Experiment 1, but received parallel instruction conditions in which they were asked for “for “something unexpected and bizarre”.

Experiment 2 also included a manipulation of sentence order such that half of the participants saw the materials in the same order as that used in Experiment 1 and the other half saw the last two sentences in reversed order: Each scenario was described in three sentences: (i) a goal-setting sentence, (ii) a sentence describing some action that was a plan-step in achieving the goal and (iii) a final sentence with some additional information about the scenario that was *not* an action on the main path to the goal. In our initial analyses, we did not find this manipulation to have any effect on responses.

Before responding to the main question, participants were asked a simple question about the scenario they had just read, to ensure that they had read and understood it. As another variable manipulation, this question referred either to the goal-setting information or the additional information. Again, we found that this manipulation had little to no effect on responses to the next question.

Participants were grouped into six conditions, the key manipulation varied the instructions used in each of the four conditions: the Unexpected (“Then, something unexpected occurred. What do you think happened?”), Unexpected-Good (“Then, something unexpected and good occurred. What do you think happened?”), Unexpected-Bad (“Then, something unexpected and bad occurred. What do you think happened?”), Unexpected-Bizarre (“Then, something unexpected and bizarre occurred. What do you think happened?”), Unexpected-Good-Bizarre (“Then, something unexpected, bizarre and good occurred. What do you think happened?”), and Unexpected-Bad-Bizarre (“Then, something unexpected, bizarre and bad occurred. What do you think happened?”).

After data collection, materials were labelled by two independent raters with a high level of agreement and any disagreements were resolved by discussion. Labelled data for each of the materials was then combined again and analysed as in the associated paper [1]. The Jupyter Notebook files in this database contain the analysis code used for the results in the paper.

### 2.3 Post-test

In the Post-Test to the first two experiments, participants rated each of the 20 material scenarios used in the first two experiments on their valence on a 7-point Likert-type scale (from 1-Very Negative to 4-Neutral to 7-Very Positive). Responses were then grouped into categories in which 1–3 = Negative, 4 = Neither, and 5–7 = Positive. We include overall frequencies for the materials as well as original responses.

### 2.4 Pre-test

For the Pre-Test to Experiment 3, we created positive and negative versions of the materials used in the prior studies, to create 20 pairs of materials (positive and negative matched pairs). Materials were edited to have positive and negative versions, matching on sentence length and topic, with only the valence changed. Each scenario followed a similar three-sentence structure as used previously, but with valence information added: scenario descriptions had (i) a goal sentence, (ii) additional information designed to convey the valence of the scenario, and (iii) a non-valenced plan-step. Participants were randomly assigned to one of four groups corresponding to a material set based on a Latin-square design. Each participant saw 20 materials, half of which were negative, and half of which were positive. Participants saw only one version of each material, so that no participant saw both the negative and the positive version of a material-pair. Participants were asked to rate each material scenario on a 7-point Likert-type scale (from 1-Very Negative to 4-Neutral to 7-Very Positive). We report the original ratings given by each participant on each material, as well as the category response.

### 2.5 Experiment 3

Ten material pairs were chosen from the pre-test to be used in Experiment 3. Materials were split into two subsets, matched on strength of positive and negative valence of their respective versions. That is, the first subset was not significantly different from the second subset in either positive or negative versions, nor were the positive and negative versions of the subsets significantly different from each other based on the ratings given in the pre-test. Participants were again randomly assigned to one of four groups corresponding to a material set based on a Latin-square design. Each participant saw 5 positive and 5 negative materials, with no participant seeing both the positive and negative version of any given material pair.

Before responding to the main question, participants were asked a simple question about the scenario they had just read, to ensure that they had read and understood it. This question only referred to the goal-setting information, and sentence order was not changed.

Participants answered the non-valenced instruction used in the previous experiments, “Then, something unexpected occurred. What do you think happened?” Responses were labelled by two independent raters on answer category, valence/sentiment, and goal-objects used in the response. Inter-rater agreement was high and any disagreements were resolved by discussion. The Jupyter Notebook files in this database contain the analysis code used for the results in the paper [1].

## Ethics Statement

Data collection from human subjects for all experiments listed was conducted with the approval of University College Dublin's ethics review board. All participants completed informed consent before participating in the studies and were allowed to discontinue participation at any time.

## CRediT Author Statement

**Molly Quinn:** Conceptualization, Methodology, Experimentation, Writing; **Kathleen Campbell:** Methodology, Experimentation; **Mark Keane:** Conceptualization, Methodology, Experimentation, Writing.

## Declaration of Competing Interest

The authors declare that they have no known competing financial interests or personal relationships which have or could be perceived to have influenced the work reported in this article.

## Data Availability

Three Datasets Reporting Unexpected Events for Everyday Scenarios (Original data) (Mendeley Data).

## Supplementary materials

Image, application 1
