# Supplementary material for: Three datasets reporting unexpected events for everyday scenarios: Over 9000 events human-labelled for overall valence/sentiment, topic category, and relationship to the initial goal of the scenario
Source: Data Brief. 2021 Mar 3;35:106935. doi: 10.1016/j.dib.2021.106935 (PMC7967011; doi:10.1016/j.dib.2021.106935)
Supplement: Supplementary file 1 [file mmc1.docx]

Variables in the Data Files

1. **Folder: 0_data/**
2. **0_Experiment1/expt1_master_post_resolve.csv**

expt – the experiment number

cond – the survey ID used in surveygizmo

var_feature1 - the instruction condition assigned to the participant (UNEXP, UNEXP-GOOD, UNEXP-BAD, or GOAL-FAIL)

user_id - participant user ID

material – material name

material_variant – the order in which the goal- and non-goal sentences were presented to the participant (goal = goal sentence last, non-goal = non-goal sentence last)

answer_text – responses written by the participants

rater_order – the order of labels in the all_ans_codes, all_senti_js and all_obj_js variables (always mk, kc, mq)

all_ans_codes – the answer category label given by the three raters separated by a space

all_senti_js – the valence label given by each of the three raters separated by a space

all_obj_js – the goal-related words label given by each of the three raters separated by a space

best_senti – the final valence label assigned after consensus

best_obj – the final goal-related words label assigned after consensus

best_ans – the final answer category label assigned after consensus

order – order of material presentation (not given here)

1. **1_Experiment2/**

This folder contains all the post-consensus material files. Materials used in Experiments 1 and 2 are attached in 3_Materials/Expt1_Materials.xlsx and materials used in Experiment 3 are attached in 3_Materials/Expt3_Materials.xlsx. Descriptions of the categories for each material are attached in Expt1_Labels.xlsx and Expt3_Labels.xlsx.

Files are set up so that each row is a participant and their response to that material. Variables consistent between material files are the following:

user_id - participant user ID

material – material name

cond – the survey ID used in surveygizmo

expt – the experiment number

var_feature1 – the valence condition assigned to the participant (UNEXP, UNEXP-GOOD, or UNEXP-BAD)

var_feature2 – the bizarreness condition assigned to the participant (Bizarre or Normal)

var_feature3 – the order in which the goal- and non-goal sentences were presented to the participant (control = goal sentence last, reverse = non-goal sentence last)

var_feature4 – which attention question was asked of the participant (goal = about the goal information, non-goal = about the non-goal information)

answer_text – responses written by the participants

neg, pos, neither – these three variables contain a 1 for agreed valence labels after consensus

non_goal_object, goal_object, both_objects, neither_object – these four variables contain a 1 for agreed goal-related word labels after consensus

1. **2_PostTest/expt1_posttest.csv**

This file contains the frequencies of participants rating each material used in Experiments 1 and 2 as positive (Pos), negative (Neg), or neither (Neither).

1. **2_PostTest/post_test_responses.csv**

This file contains the response of each participant to each material on a Likert-type scale where 1 = Very Negative, 4 = Neutral, and 7 = Very Positive.

user_id – participant user ID

cond – which order were the materials presented in (V1a = xxx, V1b = yyy)

1. **3_Experiment3/expt3_master_post_resolve.csv**

user_id – participant user ID

answer_text – participant response

expt – experiment number used as surveygizmo ID

ans_code – the final answer category label assigned after consensus

ans_count – just a check that there is only one answer category per participant per material, all 1s

val_code – the final valence category label assigned after consensus

val_count – just a check that there is only one valence label per participant per material, all 1s

material – material name

cond – valence condition of the material the participant was shown (pos_mat = positive material, neg_mat = negative material)

1. **3_Experiment3/expt3_pretest.csv**

This file contains the response of each participant to each material on a Likert-type scale where 1 = Very Negative, 4 = Neutral, and 7 = Very Positive.

user_id – participant user ID

variable – material name

value – label of Likert Scale

label – participant ratings reassigned into categorical variable

value_number - numbers of Likert Scale

category – participant ratings reassigned into categorical variable as numbers (1=negative, 2 = Neutral, 3 = Positive)

positive, negative

expected_valence – the expected valence of the designed material (pos or neg)

neg_value_number – expected negative materials’ participant ratings recoded so that more negative values are higher numbers. (i.e.; for expected positive materials 7 = Very Positive, and for expected negative materials 7 = Very Negative)

1. **Folder: 2_pipeline/**
2. **expt1_master_data.csv**

Variable names and meanings the same as 0_Experiment1/expt1_master_post_resolve.csv, except additional variables as follows:

pos, neg, neither, resolve - these variables contain a 1 for agreed valence labels after consensus

1. **expt2_master_data.csv**

Variable names and meanings the same as in **1_Experiment2/**, except additional variables as follows:

best_ans – the final answer category label assigned after consensus

value – just a check that there is only one answer category per participant per material, all 1s

best_senti – the final valence label assigned after consensus

best_obj – the final goal-related words label assigned after consensus

1. **Folder: 3_materials/**

For each material used in Experiments 1 and 2, and the materials used in Experiment 3, **Expt1_Labels.xlsx** and **Expt3_Labels.xlsx,** respectively list the answer category labels used in the *best_ans* variable in the corresponding experiment data files and the description of the category denoted by each label.
